# Contrasting the Community Structure of Arbuscular Mycorrhizal Fungi from Hydrocarbon-Contaminated and Uncontaminated Soils following Willow (*Salix* spp. L.) Planting

**DOI:** 10.1371/journal.pone.0102838

**Published:** 2014-07-17

**Authors:** Saad El-Din Hassan, Terrence H. Bell, Franck O. P. Stefani, David Denis, Mohamed Hijri, Marc St-Arnaud

**Affiliations:** 1 Botany & Microbiology Department, Faculty of Science, Al-Azhar University, Nasr City, Cairo, Egypt; 2 Biodiversity Centre, Institut de recherche en biologie végétale, Université de Montréal and Jardin botanique de Montréal, Montreal, Quebec, Canada; USDA-ARS, United States of America

## Abstract

Phytoremediation is a potentially inexpensive alternative to chemical treatment of hydrocarbon-contaminated soils, but its success depends heavily on identifying factors that govern the success of root-associated microorganisms involved in hydrocarbon degradation and plant growth stimulation. Arbuscular mycorrhizal fungi (AMF) form symbioses with many terrestrial plants, and are known to stimulate plant growth, although both species identity and the environment influence this relationship. Although AMF are suspected to play a role in plant adaptation to hydrocarbon contamination, their distribution in hydrocarbon-contaminated soils is not well known. In this study, we examined how AMF communities were structured within the rhizosphere of 11 introduced willow cultivars as well as unplanted controls across uncontaminated and hydrocarbon-contaminated soils at the site of a former petrochemical plant. We obtained 69 282 AMF-specific 18S rDNA sequences using 454-pyrosequencing, representing 27 OTUs. Contaminant concentration was the major influence on AMF community structure, with different AMF families dominating at each contaminant level. The most abundant operational taxonomic unit in each sample represented a large proportion of the total community, and this proportion was positively associated with increasing contamination, and seemingly, by planting as well. The most contaminated soils were dominated by three phylotypes closely related to *Rhizophagus irregularis*, while these OTUs represented only a small proportion of sequences in uncontaminated and moderately contaminated soils. These results suggest that *in situ* inoculation of AMF strains could be an important component of phytoremediation treatments, but that strains should be selected from the narrow group that is both adapted to contaminant toxicity and able to compete with indigenous AMF species.

## Introduction

Phytoremediation is an inexpensive and sustainable alternative to the extraction and chemical treatment of hydrocarbon-contaminated soils, but depends on the identification of plant species that a) grow efficiently in the presence of contaminants, and b) promote microbial communities that degrade hydrocarbons more rapidly than those that were present before planting. Willows (*Salix* spp.) are strong candidates for phytoremediation, as they are known to grow rapidly, even in the presence of soil contaminants [1], and have a wide growth range [2]. In addition, microbial hydrocarbon-degrading activity was recently shown to be upregulated in the rhizosphere of the Fish Creek willow cultivar (*Salix purpurea*) in hydrocarbon-contaminated soils [3]. The bioremediation potential of willows likely shifts based on the specific microbial assemblages that are promoted within the rhizosphere; we previously showed that across 11 field-tested willow cultivars, bacterial and fungal community composition varied based on contaminant concentration, and that fungi were influenced dramatically by willow identity [4].

Arbuscular mycorrhizal fungi (AMF), the nearly ubiquitous plant root symbionts, are known to improve plant health under a number of stressful conditions, including high temperatures [5], drought [6], [7], and heavy metal contamination [8]–[10]. There is also some evidence that AMF can help to increase phytoremediation of organics [11]–[13], but the mechanisms behind this activity remain unknown. A number of ectomycorrhizal fungi actively metabolize hydrocarbon contaminants [14], but AMF are generally thought to depend on plant-provided carbon. In some soils, AMF may indirectly alter soil function by modifying bacterial or ectomycorrhizal communities [15]–[17], but it is not known whether this occurs in contaminated soils.

Although plant-AMF symbioses are widespread and often mutually beneficial, organic contaminants can negatively impact this relationship, leading to decreases in AMF growth, colonization of roots, and spore production and germination [13], [18], [19]. In addition to the direct toxic effects of pollutants, AMF may receive reduced benefits from their plant partners if plants shunt carbon towards hydrocarbon-degrading microorganisms through root exudates. Plants are known to increase stimulation of hydrocarbon-degrading microorganisms in the presence of hydrocarbon contaminants [3], [20], but supplying carbon substrates to rhizosphere microorganisms can represent a substantial cost to plants, particularly in the case of AMF symbioses [21]. This suggests that tradeoffs in carbon allocation to microorganisms may be necessary in contaminated soils. Marschner et al. [22] have already demonstrated that such tradeoffs do occur; using a split-root experiment, they showed that colonization of pepper plant roots by *Rhizophagus irregularis* (formerly *Glomus intraradices*) in one pot decreased plant root exudation, which subsequently decreased *Pseudomonas fluorescens* densities in the other pot in which *R. irregularis* was absent [22]. Allocation towards hydrocarbon-degrading microorganisms, as opposed to AMF symbioses, may result in reduced plant influence over AMF community composition.

Amplicon-targeted high-throughput sequencing has been used in recent years to specifically target the community structure of AMF, in order to determine some of the environmental factors that govern the distribution of these important organisms [23]–[28]. Although AMF diversity in soils has been shown to decline in the presence of metal contaminants [29], it is unknown how sensitive these communities are to hydrocarbon contaminants. Here, we show the results of a 454-pyrosequencing analysis of rhizosphere soil samples taken from within a phytoremediation field trial established at the site of a former petrochemical plant. We expected that the presence of hydrocarbon contaminants would strongly influence the AMF community that is available for recruitment by introduced willows. While hydrocarbon contaminants had a strong effect on AMF community structure, the composition of AMF communities was not consistently related to specific willow cultivars. However, willow planting appeared to increase the abundance of the most dominant OTU, possibly due to opportunistic associations between dominant AMF and the introduced plants, which furthers the idea that AMF distribution patterns can be somewhat stochastic and dependent on dispersal limitations [30], [31].

## Materials and Methods

### Ethics statement

No specific permits were required for the described field study. The land on which we conducted the phytoremediation field is privately owned by ConocoPhillips. This field study did not involve endangered or protected species.

### Experimental design

Our sampling occurred in the context of a phytoremediation field trial at the site of a former petrochemical plant in Varennes, QC, Canada (45°43 N, 73°22 W). A description of the site, design, soil sampling, and DNA extraction has been described previously [4]. Planting of fresh willow clippings occurred on 6–7 June 2011. Once planted, clippings develop roots and shoots on site, and are direct clones of the progenitor plant. The ∼5000 m^2^ site is separated into two areas, in which we designated 3×300 m^2^ blocks for each; one area is contaminated primarily with petroleum hydrocarbons as a result of previous petrochemical refining activities (Blocks C3, C4 and C5) and an adjacent area remains uncontaminated (Blocks N1, N3 and N5; an initial survey in 2010 found that petroleum concentrations in this area are below the detection limit). Within each block are 12 randomly assigned 25 m^2^ square plots that were each planted with one of 11 willow cultivars or left as unplanted controls. The willow cultivars used were Fish creek (*S. purpurea*), SX67 (*S. miyabeana*), SX61 (*S. sachalinensis*), S05 (*S. nigra*), S25 (*S. eriocephala*), S365 (*S. caprea*), SV1 (*S. dasyclados*), S54 (*S. acutifolia*), S44 (*S. alba*), S33 (*S. viminalis*), and Millbrook (*S. purpurea* x *S. miyabeana*). More details on the origin and characteristics of these cultivars are provided in Bell et al. [4]. Within each planted plot are five rows of 15 plants, with 30 cm of spacing between each plant. A 1 m margin was left unplanted on either side of each row. Mean temperature from planting to soil sampling (6 June 2011–8 August 2011) was 21.9°C, with 196.9 mm of total precipitation (Environment Canada–Verchères Weather Station; http://www.climate.weatheroffice.ec.gc.ca/index.html).

Soil sampling occurred on 6 August 2011 for the uncontaminated blocks and on 8 August 2011 for the contaminated blocks. Within each plot, five rhizosphere soil samples were collected from randomly selected trees that were destructively harvested and pooled. Roughly 100 g of rhizosphere soil from the top ∼0–15 cm was collected from each tree by shaking off excess soil and only collecting soil that remained adherent to roots. Similarly, five bulk soil samples were collected from the top 15 cm of soil and pooled in the unplanted control plots. We flash-froze approximately 50 g of each pooled sample on-site in dry ice and ethanol, and stored these samples in 50-ml Falcon tubes at −80°C prior to DNA extraction.

Twelve soil samples from each contaminated block were sent to Maxxam Analytics (Montreal, Quebec, Canada) on 9 August 2011, for F1–F4 hydrocarbon analysis (equivalent to the sum of all aromatic and aliphatic hydrocarbon compounds with chain lengths of C6–C50) according to the standard protocol set forth by The Canadian Council of Ministers of the Environment (http://www.maxxam.ca/solutions/sol_env_CCME_Petr_Hydroc_0805.pdf). Other soil characteristics were determined from a pooled sample for each block at Agridirect Inc (Longueuil, Quebec, Canada) according to their standard operating procedures. Hydrocarbon concentrations and soil parameters were reported previously [4]. Mean F1–F4 hydrocarbons for each contaminated block were 709 mg kg^–1^ (±339 s.e.) in block C3, 2143 mg kg^–1^ (±551 s.e.) in block C4 and 3590 mg kg^–1^ (±760 s.e.) in block C5.

### DNA extraction, amplification and 454-pyrosequencing

We extracted total soil DNA from each of the 72 samples using the MoBio PowerSoil DNA Isolation Kit (MoBio Laboratories, Carlsbad, CA, USA). We used a nested PCR approach to target AMF-specific sequences from a ∼800-bp stretch of 18S rDNA. A first amplification was performed with the primers NS1 (5′-GTA GTC ATA TGC TTG TCT C-3′) and NS41 (5′-CCC GTG TTG AGT CAA ATT A-3′) [32] and a second with AML1 (5′-ATC AAC TTT CGA TGG TAG GAT AGA-3′) and AML2 (5′-GAA CCC AAA CAC TTT GGT TTC C-3′) primers [33] containing unique multiplex identifier (MID) tags for each sample. PCR reagent concentrations are as described in Bell et al. [4] using 50 µl volumes. The first round of amplification was performed using the following cycling conditions: 3 min at 95°C, 30 cycles of 1 min at 95°C, 1 min at 50°C, and 1 min at 72°C, with a final elongation step of 15 min at 72°C. For the second round of amplification, 1 µl of product was spiked into the reaction mix, and cycling conditions were: 3 min at 95°C, 30 cycles of 45 sec at 95°C, 45 sec at 58°C, and 45 sec at 72°C, with a final elongation step of 15 min at 72°C. Amplicons were pooled in equimolar ratios, which were then sent for sequencing. After a number of trials, we could not amplify sequences from two samples in block C5 (Cultivars S25 and S54), and these were omitted from amplicon pools. Sequencing was performed using 454 GS FLX+ chemistry (Roche, Branford, CT, USA). To increase nucleotide complexity in the sequencing reaction and thus avoid the synchronized flow signal that can be associated with amplicon sequencing using this chemistry, we truncated 1/3 of our MID codes by 1 base and 1/3 by 2 bases [34]. The sequence data generated in this study were deposited in the NCBI Sequence Read Archive and are available under the project number SRP043030.

### Sequence quality processing and phylogenetic analysis

Our sequence processing pipeline was carried out using primarily Mothur v.1.32.1 (available at http://www.mothur.org/), following the 454 SOP outlined in Schloss et al. [35]. We split our.sff files into.fasta and.qual files using ‘sffinfo’, and sequences were then binned by MID and quality filtered with ‘trim.seqs’ using the following parameters: maxambig = 0, maxhomop = 8, bdiffs = 1, pdiffs = 2, qwindowaverage = 30, qwindowsize = 50, minlength = 400. Sequences were reduced to only those that were unique using ‘unique.seqs’, after which they were aligned to the Mothur-interpreted Silva eukarotic database using ‘align.seqs’ (ksize = 9, align = needleman, gapopen = −1). Aligned sequence length was reduced to only the overlapping region using ‘screen.seqs’ (start = 5406, optimize = end, criteria = 95) and ‘filter.seqs’ (vertical = T, trump = .). We further reduced sequencing errors using ‘pre.cluster’ (diffs = 4) and ‘chimera.uchime’ prior to clustering. In addition, we selected only those sequences that corresponded to AMF by combining ‘classify.seqs’ (using the Silva eukaryotic database plus the AMF 18S rRNA database described in Krüger et al. [36]) and the ‘get.lineage’ command (taxon = *Eukaryota*; *Fungi*; *Glomeromycota*) prior to calculating the distance matrix, in order to select only those sequences that corresponded to AMF. We then created a distance matrix using ‘dist.seqs’ and formed OTUs using average-neighbour clustering with the command ‘cluster.split’.

While the primers we used favour AMF amplification, they also amplify 18S rDNA sequences from other phyla (Figure S1). This was a problem for some samples, especially those originating from highly contaminated soils. Prior to the ‘get.lineage’ step, we had a mean of 1475 sequences per sample. Following the selection of sequences that classified as *Glomeromycota*, 2.32–80.56% of sequences were lost from each sample taken from the uncontaminated blocks (mean 16.11±15.94 (SD) %), 2.17–55.47% of sequences were lost from each sample taken from contaminated block C3 (mean 11.68±15.66 (SD) %), and 1.54–100.00% of sequences were lost from each sample taken from blocks C4 and C5 (mean 77.16±35.85 (SD) %). Determination of OTUs was then performed using ‘dist.seqs’ and ‘cluster.split’, and OTUs were calculated to 97% similarity.

Since identification of AMF OTUs based on simple BLASTing of the first half of 18S rDNA sequences can lead to ambiguities (i.e. multiple hits with different taxon IDs), a Bayesian phylogenetic analysis was performed on an alignment that included the consensus sequences for each OTU, the sequences from Krüger et al. [36], and the closest matches recovered from the MaarjAM database [37]. Sequences were aligned using MUSCLE v.3.5 [38]. The DNA substitution model was determined using the Bayesian information criterion calculations implemented in jModelTest v2.1.3 [39]. Bayesian phylogenetic analyses were performed using the parallel version of MrBayes v3.1.2 [40], [41]. The value of the temperature parameter for heating chains was adjusted to keep the acceptance rate of swaps between 10 and 70%. Two simultaneous and independent runs were evaluated for each analysis. Convergence of MCMC chains and the values of effective sample size of parameters were assessed with Tracer v.1.5 (http://tree.bio.ed.ac.uk/software/tracer). The number of trees saved was set to 15 000 and the first 3000 trees were excluded before computing the consensus tree with Bayesian posterior probabilities.

### Statistical analysis

We tested the effect of contaminants on AMF phylogenetic community structure at three different hydrocarbon levels: not contaminated (NC; blocks N1, N3, N5), low contamination (LC; block C3), and high contamination (HC; blocks C4, C5). We separated the blocks as such due to large differences in contaminant concentration and community response that were not known *a priori*. To test for differences in phylogenetic structure, we calculated the mean abundance of each AMF OTU for each hydrocarbon level, and compared the phylogenetic structure of each by computing the standardized effect size of the mean pairwise phylogenetic distance (*SES_MPD_*). The mean pairwise distance was weighted by OTU abundance, and the observed phylogenetic relatedness was compared with null communities that were generated by randomizing the AMF community data matrix abundance within OTUs (1000 iterations). Calculations were done using the ‘picante’ package [42] in R v.3.0.2. (R Foundation for Statistical Computing; available at http://www.R-project.org). OTU composition between contaminant levels was also tested for significance using the unweighted metric in FastUnifrac [43].

For comparative analyses across all plots, the number of sequences per sample was subsampled to 98 sequences, due to the low number of AMF-specific sequences in some samples. Redundancy analysis was performed using the ‘rda’ function from the ‘vegan’ package in R, and constraining AMF OTU composition by contaminant level and willow cultivar identity. To abundance of the dominant AMF OTU in each sample was calculated from the OTU matrix produced following sequence processing.

## Results and Discussion

### Phylogenetic identification of AMF OTUs

**Figure 1:**
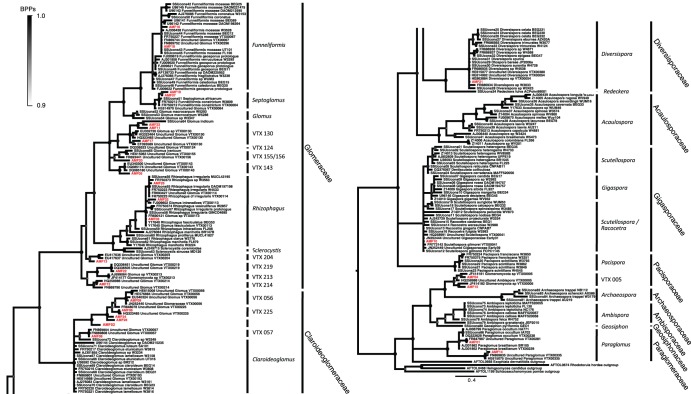
Bayesian phylogenetic tree based on nuclear small subunit (SSU) rDNA consensus sequences showing the distribution of the 27 OTUs recorded in Varennes (red labels) among the *Glomeromycota* tree. Sequence data were analysed with the SSU sequences (black labels) from Krüger et al. (2012) and the closest match recovered from MaarjAM database. Circles on nodes indicate Bayesian posterior probabilities ranging from 0.9 (white) to 1 (black). Node size also decreases with decreasing probabilities. The scale represents the branch length corresponding to expected substitutions per site.

After quality filtering, a total of 69 282 AMF-specific sequences were recorded across the three soil types, and these represented 27 OTUs at a similarity threshold of 97%. Only OTUs with a cumulative abundance greater than 0.5% for the three treatments were considered. The Bayesian phylogenetic analysis (Figure 1) shows that these 27 OTUs were members of the *Archaeosporaceae* (VTX 005), *Claroideoglomeraceae*, *Diversisporaceae*, *Gigasporaceae*, *Glomeraceae*, and *Paraglomeraceae*. The *Glomeraceae* had the highest number of assigned OTUs, with nine OTUs representing four different genera (*Funneliformis, Septoglomus, Glomus* and *Rhizophagus*) and three virtual taxa (VTX 130, VTX 156, VTX 143). The six *Claroideoglomeraceae* OTUs were all related to virtual taxa, and clustered on a separate sister clade to the *Claroideoglomus* genus. The lack of described taxa in this clade implies that these OTUs likely represent AMF taxa that do not sporulate often, if at all. At the other extreme, *Diversispora* and *Scutellospora/Racocetra* were each represented by just a single OTU.

The identified richness at Varennes is relatively high when compared to records from natural and agricultural soils, especially given that only willows were sampled as host plants. For instance, Verbruggen et al. [44] recorded 1 to 11 AMF taxa by T-RFLP analysis of 25S rDNA fragments across 40 agricultural soils, and 12 AMF OTUs were observed by Hassan et al. [45] in soils that had undergone long-term fertilizer application. Öpik et al. [23] recorded 47 AMF taxa in a boreonemoral forest by 454-sequencing, but sampled root tissues from 10 host plants. Similarly, only a small number of AMF species have been identified in studies that have surveyed AMF diversity in hydrocarbon-contaminated soils. Cabello [19] only observed spores that identified as *Gigaspora* sp., *Glomus aggregatum* and *Funneliformis mosseae* (formerly *Glomus mosseae*) in trap cultures, and Fester [46] identified OTUs closely related to *R. irregularis* and *F. mosseae* in a constructed wetland. Franco-Ramirez et al. [47] reported seven AMF morphospecies from the roots of *Echiniclea polystachya* and two *Citrus* species that were growing in soil that was chronically contaminated with polyaromatic hydrocarbons. With respect to the diversity of AMF in metal-polluted soils, less than 10 species are generally reported [29], [48]–[50], with the exception of 14 AMF small subunit (SSU) types recorded in the roots of *Solidago gigantea* [51]. In surveys of soils contaminated with trace metals, *F. mosseae* has been reported as an abundant AMF taxon [29], [52]. Here, two OTUs clustered with *F. mosseae* sequences, but the relative abundance of these was below 1.5% at each contaminant level.

### Influence of hydrocarbon contaminants on AMF community structure

**Figure 2:**
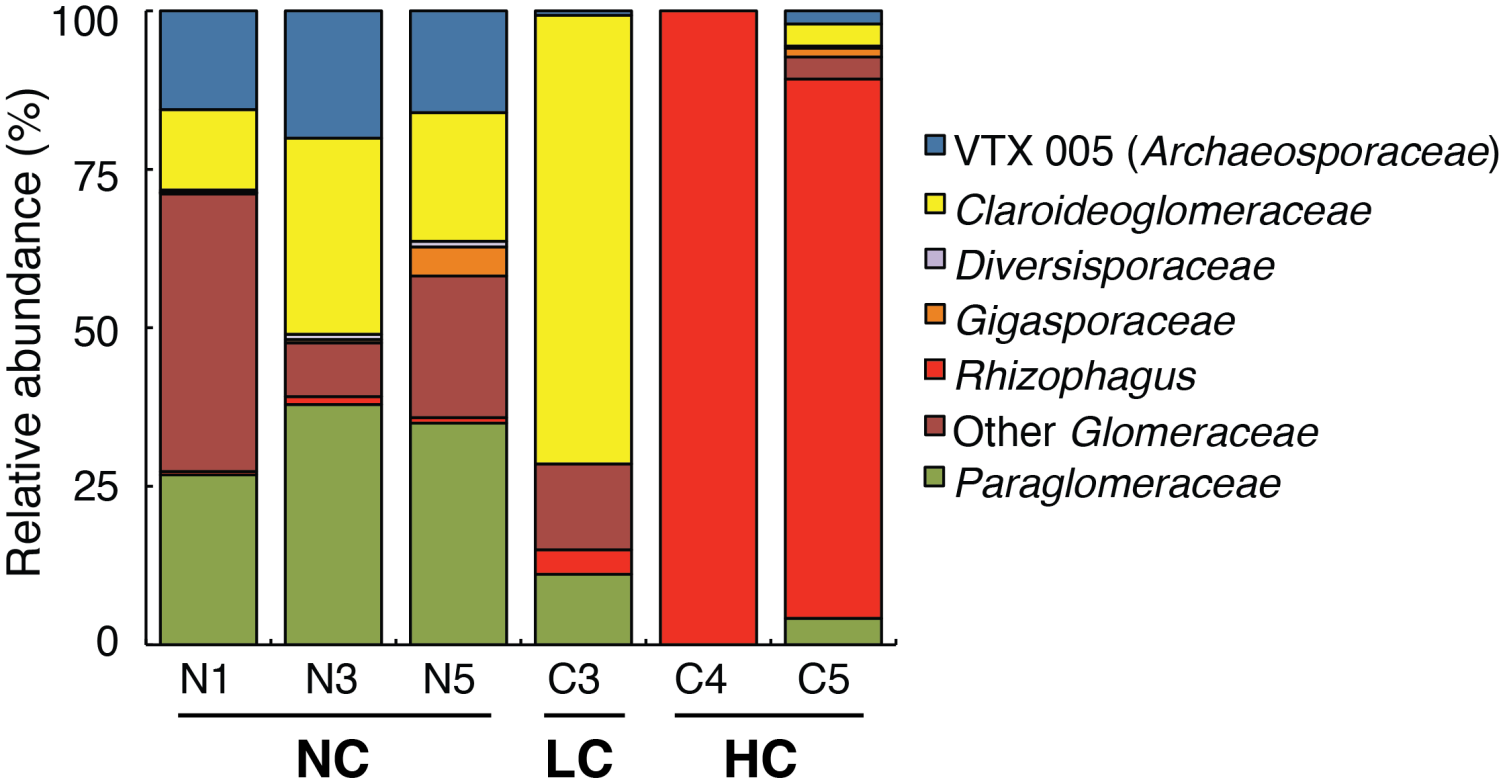
Mean relative abundance of AMF families from all samples in the uncontaminated blocks (N1, N3, N5; n = 12 per block), the moderately contaminated block (C3; n = 12), and the highly contaminated blocks (C4, C5; n = 3 per block). Labels underneath block numbers indicate no hydrocarbon contamination (NC), low hydrocarbon contamination (LC), and high hydrocarbon contamination (HC).

**Figure 3:**
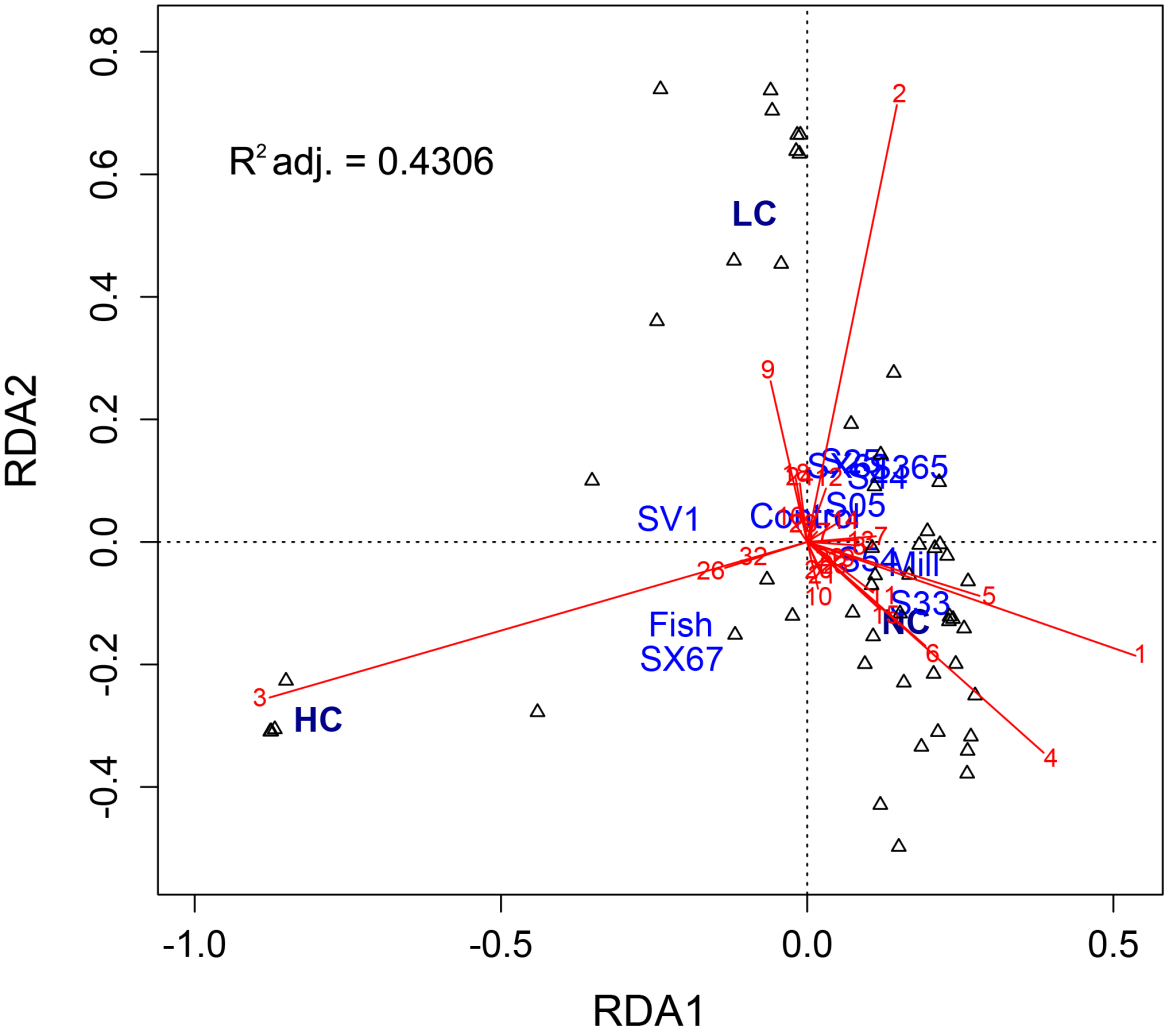
Redundancy analysis (RDA) showing the relationship between contaminant level, willow cultivar identity, and the abundance of AMF OTUs. The adjusted R^2^ value indicates the amount of variance in AMF community composition accounted for by the constraining variables (contaminant level and willow identity). The location of contaminant level and cultivar labels represent factor centroids. Red numbers indicate AMF OTUs, as shown in Figure 1. Black open triangles represent individual samples.

The richness of AMF OTUs appeared to be moderately affected by the presence of hydrocarbons in LC and HC soils, as 19 and 17 OTUs were observed, respectively, compared with 24 in NC soils. In the HC blocks, only four OTUs (AMF01, AMF03, AMF04, AMF26) had a relative abundance above 1%. The relative abundance of AMF strains shifted dramatically between NC/LC and HC soils (Figure 2) and to a lesser extent between NC and LC soils. When examining mean phylogenetic structure for each contaminant level, the calculation of standard effect size of the mean pairwise distance (Table 1) showed that AMF phylogenetic structure was random for the NC and LC soils, but clustered in HC soils. This is mainly explained by the extremely high relative abundance of AMF03 in HC soils (87.6% on average; close match to *R. irregularis*), while this OTU represented ∼3.7% of sequences on average in LC soils and <1% of sequences on average in NC soils. Additionally, AMF26 and AMF32 clustered within the *Rhizophagus* clade, and combined, these three OTUs represented 89% of all reads in HC soils. The strong effect of hydrocarbon contaminants was confirmed by RDA analysis (Figure 3). An abundance of AMF01 and AMF04 was characteristic of NC soils, while a high abundance of AMF02 and AMF03 was characteristic of LC and HC soils, respectively.

**Table 1:** Measures of the AMF phylogenetic structure recorded across soils with no hydrocarbon contamination (NC), low hydrocarbon contamination (LC), and high hydrocarbon contamination (HC).

Soil^1^	SES_mpd_ 2	*P*-value	Phylogenetic structure
NC	0.17	0.52	Random
LC	0.26	0.56	Random
HC	−5.94	0.0009	Clustered

1 NC: not contaminated; LC: low contamination; HC: high contamination.

2 Standard effect size of the mean pairwise distance.

Chagnon et al. [53] describe the *Glomeraceae* as a ruderal group (i.e. one that is quick to colonize disturbed areas), which may particularly benefit from allocating more biomass to plant roots than to surrounding soil, as it limits damage from external disturbances. The increase in *Rhizophagus* in HC soils corresponds with this description, but other members of the *Glomeraceae* did not thrive when contaminants were present. The low abundance of *Rhizophagus* in NC and LC soils (in particular, AMF03, which was recorded within all 6 blocks) indicates that its high abundance in HC plots is not simply related to primer/methodological bias. The dominance of strains related to the *Rhizophagus* clade is somewhat unexpected, since previous *in vitro* studies demonstrated significant negative effects of hydrocarbons on the development of strains identified as *R. irregularis* DAOM 197198 [13], [18], [54]–[56]. In this study, however, none of the OTUs recorded within the *Rhizophagus* clade (AMF03, AMF26, AMF32) was homologous with the SSU sequence of *R. irregularis* DAOM 197198, suggesting the existence of genetic variants, or ecotypes, of *R. irregularis* that are more tolerant to hydrocarbons. Koch et al. [57] demonstrated that the presence of genetically different *R. irregularis* isolates affected host-plant fitness, and this emphasizes the ecological importance of within-isolate genetic variation. Greenhouse and *in vitro* testing of *Rhizophagus* strains from the Varennes site is the next step in demonstrating their potential for accelerating bioremediation of hydrocarbons, although such approaches will not reveal how the activity of these strains is modified by biotic and abiotic factors *in* *situ*.

The prevalence of the *Rhizophagus* clade in HC soils was surprising, given that 21 and 19 AMF OTUs were identified in rhizosphere soils taken from *Eleocharis obtusa* and *Panicum capillare*, respectively, plants that were growing spontaneously in highly contaminated sediments near our HC blocks [58]. However, this may be explained by host plant differences. Another species of willow, *Salix repens*, has been shown to support a much higher proportion of ectomycorrhizal than AM fungal symbionts, particularly when compared with co-occurring non-woody plants, despite receiving substantial growth benefits from AMF partners [59], [60]. In addition, our sampling occurred only 2 months after planting, which may have limited interactions between willows and resident AMF species, as the dominance of AMF species is known to change both seasonally and as a product of succession [61]–[63]. However, van der Heijden et al. [60] found that total AMF colonization of *S*. *repens* was equal after 12, 20 and 30 weeks, suggesting that AMF saturation of roots does not necessarily require long periods of time.

Although the high abundance of *Rhizophagus* in HC soils indicates increased resistance to high hydrocarbon concentrations, this alone did not provide these strains with a growth advantage when only moderate amounts of contaminant were present. Although we might have expected highly hydrocarbon-tolerant strains to increase steadily in abundance with increasing contaminant concentrations, *Claroideoglomeraceae* was the dominant group in LC soils, while the relative abundance of taxa related to *Glomeraceae* did not increase with respect to NC soils (Figure 2). The proportion of *Glomeraceae* that classified as *Rhizophagus*, however, did increase marginally. This suggests that hydrocarbon-tolerant members of the *Claroideoglomeraceae* are superior competitors. This was also observed by de la Providencia et al. [58], who grew leeks under greenhouse conditions in order to trap AMF from the above-mentioned hydrocarbon-contaminated sediments collected near our HC blocks. They found that while *Claroideoglomeraceae* represented 20 to 25% of sequences in the field, the relative abundance of this family ranged from 33 to 86% under greenhouse conditions. This strong competitive advantage suggests that if highly hydrocarbon-tolerant *Rhizophagus* isolates are identified as desirable plant partners for the phytoremediation of hydrocarbons, it may be challenging to promote these associations when contaminant concentrations are low.

**Figure 4:**
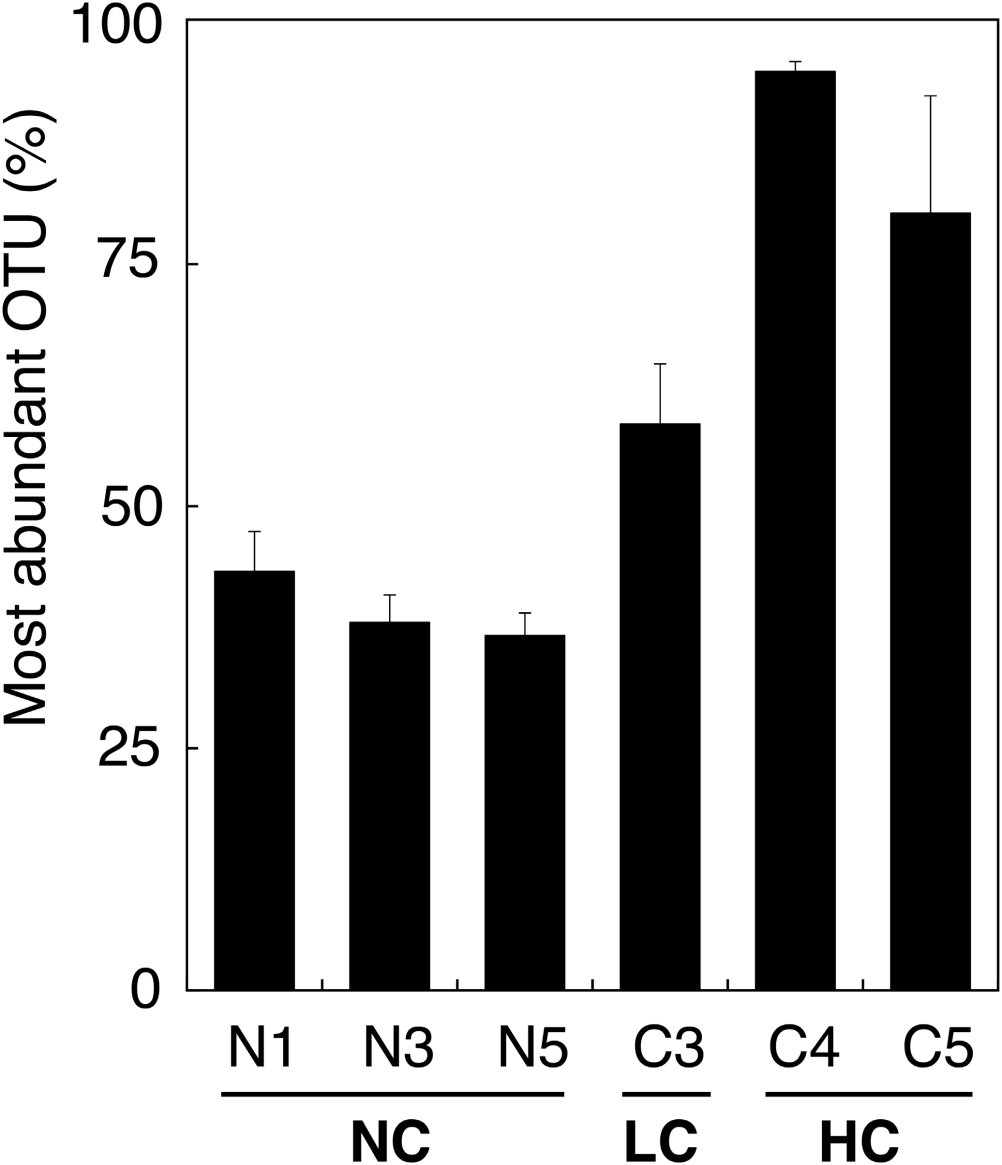
Mean relative abundance of most dominant OTU across blocks. Error bars indicate standard error. Labels underneath block numbers indicate no hydrocarbon contamination (NC), low hydrocarbon contamination (LC), and high hydrocarbon contamination (HC).

Dumbrell et al. [30] previously showed that AMF species abundance distributions are atypical of those observed for many other groups of organisms, in that the most abundant OTU/species occupies an extremely high proportion of the community (on average ∼40%). This was our observation as well, particularly as contamination increased (Figure 4). A decrease in diversity with increasing contamination is not unusual for microbial communities [64]–[66], but it was surprising to observe that AMF communities from HC soils were almost entirely dominated by a single OTU. This again contrasts with what was observed for spontaneous vegetation near the HC plots, in which the most abundant OTUs represented ∼25% of all AMF sequences [58].

### Influence of willow introduction on AMF community structure

We anticipated that there would be a strong effect of willows on AMF community structure, since willows are known to rapidly form associations with AMF [60], [67], and directly associate with AMF in many different soil environments [59], [68]–[70]. However, our previous observation of a relationship between fungal community composition and willow phylogeny in the HC soils [4] was not replicated by AMF assemblages. Our redundancy analysis demonstrates that contaminant level had a much stronger effect on AMF community composition than willow identity (Figure 3). Only 3 of 11 cultivars (Fish Creek, SV1, SX67) yielded sufficient AMF-specific sequences to examine OTU distribution, but these represented willows from both the phylogenetic cluster that strongly promoted *Pezizomycetes* (Fish Creek), and from the other, which did not (SV1, SX67). With the exception of SX67 in block 5, at least 96% of AMF-specific sequences classified as *Rhizophagus*. An initial survey in 2011 indicated that soil hydrocarbon concentrations for the plots in which these cultivars were planted were among the five lowest for the HC blocks, along with the plots for cultivars S44 and S54 (∼1400 mg kg^−1^ on average, to a maximum of 2449 mg kg^−1^ for SV1 in block 5; data not shown), which may indicate that AMF colonization is impeded beyond a certain contaminant threshold. However, the fact that AMF sequences were not observed within S44 and S54 samples, but were observed within both blocks for Fish Creek, SV1, and SX67 suggests an increased influence of these plants over AMF communities.

Previous reports have suggested that AMF community composition may be dependent on stochasticity and priority effects [26], [30], [71]; in other words, those species that are abundant within the rhizosphere of plants may be at least in part dependent on the relative abundance of AMF prior to plant arrival, as determined by semi-random mechanisms such as dispersal. Vierhilig et al. [72] showed that establishment of any of three distinct AMF species in the roots of barley prevented secondary colonization by *F. mosseae*, suggesting that plants may play a role in perpetuating patchy distributions of AMF. Other studies, however, have observed plant-specific AMF community composition [73], although AMF composition is generally thought to be specific to functionally related plants rather than actual plant species [25], [74]. Stochasticity appeared to play a role in our NC plots, as five different OTUs were identified as the most abundant across the 36 samples (Table S1), and this was often not consistent across replicates of the same willow cultivar. Since plant phytochemical production can shift dramatically during plant development [75] and across plant cultivars [76], communities of slow-growing AMF may not be able to respond as rapidly to plant-elicited signals as other microorganisms, leading to an increased importance of priority effects in the early stages of growth for certain plants.

Nevertheless, willow planting did seem to influence AMF communities. The most abundant OTU in the unplanted NC control plots represented 25–31% of all sequences, but increased in most planted plots, up to a maximum of 69.4% (Table S1). As suggested by Dumbrell et al. [30], this effect may be the result of increased interspecific competition, likely as a result of limited root colonization space. This could be especially pertinent in the early stages of willow growth, as root biomass is low and early colonizers would be capitalizing on an essentially vacant niche. In the LC plots, the effect was less universal, although the dominant AMF OTU in 7/11 cultivars was 27–45% higher than in the unplanted control (Table S1). Since these willows were introduced to our study site, the relationships between their newly formed roots and resident AMF may lack specificity, and this increase in abundance by the dominant OTU may simply represent opportunism by the fungus, the willow, or both.

## Conclusions

High-throughput sequencing facilitates an understanding of the ecological processes that occur during phytoremediation, as we can now examine plant-associated microbial communities in depth, while screening across a wide variety of plant and soil types [77]. For the first time, this study uses high-throughput sequencing to reveal the structure of AMF communities associated with plants that have been introduced to hydrocarbon-contaminated soils, providing an in-depth view of the changes that occur as a result of both hydrocarbon contamination and plant introduction. These results, along with previously published studies on AMF communities, suggest that both priority effects and soil conditions ultimately determine which AMF dominate specific soils. If this is the case, pre-inoculation of candidate phytoremediator plants with AMF could be a successful strategy for ensuring that specific members of this group are favoured *in situ*, although inoculated species will need to be selected from the limited group of AMF that are able to remain competitive at a given contaminant concentration. Verbruggen et al. [31] list three critical factors that will determine the survival of inoculated AMF strains in soils: the ability to survive in the target environment, niche availability and priority effects. Because plants are generally introduced to soils to initiate phytoremediation, both niche availability and priority effects can be managed, leaving only the task of identifying AMF strains that are both contaminant tolerant and sufficiently competitive to dominate the root/rhizosphere environment. Studies in which the identity of dominant AMF strains is controlled (i.e. through inoculation, or otherwise) will also help to determine whether there is an important role for AMF in shaping the composition and activities of rhizosphere bacteria and other fungi at contaminated sites, and whether this ultimately influences rates of contaminant reduction.

## Supporting Information

Figure S1 **Taxonomy of sequences not classified as Glomeromycota.**
(EPS)

Table S1 **The identity of the most abundant OTU in each sample, followed by its percent relative abundance.**
(XLSX)
